# A study on endonuclease BspD6I and its stimulus-responsive switching by modified oligonucleotides

**DOI:** 10.1371/journal.pone.0207302

**Published:** 2018-11-26

**Authors:** Liudmila A. Abrosimova, Anzhela Yu. Migur, Elena A. Kubareva, Timofei S. Zatsepin, Aleksandra V. Gavshina, Alfiya K. Yunusova, Tatiana A. Perevyazova, Alfred Pingoud, Tatiana S. Oretskaya

**Affiliations:** 1 Department of Chemistry and A.N. Belozersky Institute of Physico-Chemical Biology, M.V. Lomonosov Moscow State University, Moscow, Russia; 2 Skolkovo Institute of Science and Technology, Skolkovo, Moscow region, Russia; 3 Institute of Theoretical and Experimental Biophysics of Russian Academy of Sciences, Pushchino, Moscow region, Russia; 4 Institute of Biochemistry, Justus-Liebig University, Giessen, Germany; New England Biolabs Inc, UNITED STATES

## Abstract

Nicking endonucleases (NEases) selectively cleave single DNA strands in double-stranded DNAs at a specific site. They are widely used in bioanalytical applications and in genome editing; however, the peculiarities of DNA–protein interactions for most of them are still poorly studied. Previously, it has been shown that the large subunit of heterodimeric restriction endonuclease BspD6I (Nt.BstD6I) acts as a NEase. Here we present a study of interaction of restriction endonuclease BspD6I with modified DNA containing single non-nucleotide insertion with an azobenzene moiety in the enzyme cleavage sites or in positions of sugar-phosphate backbone nearby. According to these data, we designed a number of effective stimulus-responsive oligonucleotide inhibitors bearing azobenzene or triethylene glycol residues. These modified oligonucleotides modulated the functional activity of Nt.BspD6I after cooling or heating. We were able to block the cleavage of T7 phage DNA by this enzyme in the presence of such inhibitors at 20–25°C, whereas the Nt.BspD6I ability to hydrolyze DNA was completely restored after heating to 45°C. The observed effects can serve as a basis for the development of a platform for regulation of NEase activity *in vitro* or *in vivo* by external signals.

## Introduction

Selective formation of single-strand breaks in DNA (DNA nicking) occurs during replication [1,2], recombination [3], transcription [4–6], and repair [7,8]. More than 6000 DNA-nicking enzymes (called nicking endonucleases, NEases) have been predicted and presented in the REBASE list: http://rebase.neb.com/cgi-bin/azlist?nick. Some NEases can be a part of heterodimeric restriction endonucleases (REases) [9,10]. NEases have been used in assays for effective detection of genomic DNA [11], cancer biomarkers [12], proteins [13], and other bioanalytes [14–16]. REases and NEases fused with TALE domains [17,18], zinc-finger domains [19], along with CRISPR-Cas9 systems [20] have been used for genome editing. Nevertheless, selective cleavage of the genome at certain sites may be accompanied by undesirable off-target cleavage. Minimization of off-target effects in genome engineering is still one of the main issues for broad practical applications of the CRISPR-Cas9 system [21–23]. Numerous approaches were developed to overcome this problem, including improved mutant Cas9, modified guide RNAs (gRNAs) and fused proteins [24,25]. Among them TALE and catalytically inactive Cas9 fusions with NEases [17,18] and restriction nucleases [26,27] demonstrated improved selectivity in genome editing.

There are known examples of selective regulation of endonuclease activity using various external signals [28–31]. Among the proposed approaches, we would like to highlight structural modulation of modified DNA [32,33] or proteins [30,31,34,35] by light irradiation or changing the temperature. Oligodeoxyribonucleotides bearing non-nucleoside insertions have been used in studies on REases EcoRII, SsoII and EcoRI [36–38], T4 DNA ligase [39], some polymerases [40,41], and nucleotide excision repair enzymes [42]. The more attractive instruments are oligodeoxyribonucleotides with an azobenzene (AB) moiety attached to a non-nucleoside D-threoninol backbone (AB-insertion, S1 Fig), which can change duplex stability under UV irradiation. Under visible light, AB has a *trans*-configuration, and therefore AB intercalates between DNA bases [43] and does not disturb the DNA duplex as confirmed by NMR studies [44]. For an 8-bp DNA duplex, AB residues in the *trans*-configuration even increase thermal stability [45]. After UV light irradiation, AB switches from the *trans*- to *cis*-configuration that results in local distortion of the DNA duplex [46]. This feature can be used for the regulation of activities of DNA-binding enzymes [47]. Previously, the approach to switching an enzymatic activity with UV light by means of modified DNA duplexes has been described for T7 RNA polymerase [48].

Nevertheless, the development of systems for stimulus-responsive regulation of enzymatic activity is possible only for well-characterized proteins because the tertiary structure of a free protein and of the protein in complex with a DNA substrate are the key factors for effective design of the system. For most of NEases, the characteristic features of interactions with DNA are still not clearly understood. This study is focused on the heterodimeric REase BspD6I (R.BspD6I) from thermophilic *Bacillus* species D6 strain that has optimal functional temperature at 55°C [49]. R.BspD6I consists of a large subunit–NEase BspD6I (Nt.BspD6I)–and a small subunit (ss.BspD6I). Crystal structures are available only for free Nt.BspD6I and ss.BspD6I (PDB codes 2ewf and 2p14, respectively) without DNA substrates [50]. Previously, we have studied functional interdependence between two R.BspD6I subunits using unmodified DNA substrates and 6-methyl-2′-deoxyadenosine containing DNA [51]. Using catalytically deficient D456A and E418A variants of Nt.BspD6I, we demonstrated that the small subunit is active only in the presence of catalytically active Nt.BspD6I [52]. Thus, the conformation of DNA-bound Nt.BspD6I initiates the binding of the small subunit to a preformed DNA–protein complex followed by hydrolysis of the bottom strand of DNA. Here we present the further experiments on R.BspD6I–DNA interactions using various stimulus-responsive modified DNAs. First, we studied binding and cleavage of duplexes with AB-insertions by R.BspD6I subunits. Second, we applied modified DNA duplexes to stimulus-responsive regulation of the Nt.BspD6I activity by changing either lighting or temperature.

## Materials and methods

### Protein expression, purification, and oligodeoxyribonucleotide synthesis

Nt.BspD6I and ss.BspD6I were expressed and purified separately following the protocols described in refs. [51,53,54]. Modified oligodeoxyribonucleotides were synthesized by the standard solid-phase approach using commercially available phosphoramidites (according to the protocols provided by manufacturers), then purified by reverse-phase high-performance liquid chromatography (RP-HPLC) and characterized as described earlier [55]. Unmodified oligonucleotides were purchased from Syntol (Russia).

### Radioactive labeling of oligonucleotides

The ^32^P label was introduced at the 5′-end of an oligonucleotide (10 pmol) using T4 polynucleotide kinase (5 U, Thermo Fisher Scientific, USA) and (γ-^32^P)ATP (0.4 MBq) in a buffer consisting of 50 mM Tris-HCl (pH 7.6), 10 mM MgCl_2_, and 5 mM DTT at 37°C for 30 min. Labeled oligonucleotides were purified on columns MicroSpin G-50 (GE Healthcare, USA). Radioactivity was measured on a Tracor Analytic Delta 300 (ThermoQuest/CE Instruments, USA).

### Determination of thermal stability of DNA duplexes

To anneal the DNA duplexes (Tables 1 and 2), solutions of complementary single-stranded oligonucleotides in a buffer consisting of 10 mM Tris-HCl (pH 7.8), 150 mM KCl, and 10 mM MgCl_2_ were heated at 90°C for 5 min and then were slowly cooled down to the room temperature. Thermal stability of the duplexes (0.4–0.5 μM) was determined from the dependence of the solution’s optical density on temperature. The measurements were performed in triplicate on a U-2800A spectrophotometer (Hitachi, Japan) equipped with an SPR-10 temperature regulator, in 1 cm quartz cuvettes (Hellma, Germany) at 260 nm. DNA duplexes were incubated at 15°C for 10 min and then heated to 65°C during 100 min. Melting temperatures of the DNA duplexes were calculated as a maximum of *f'*(T) = ΔA_260_/(ΔT); the standard error did not exceed 1°C.

**Table 1:** Interaction of Nt.BspD6I and ss.BspD6I with the DNA duplexes I and I-A to I-F.

N	DNA duplex	T_m_, ± 1°C	Apparent *K*_d_ of the complex Nt.BspD6I-DNA, nM	Initial cleavage rate, nM/min, Nt.BspD6I	Relative cleavage extent, % (30 min, 37°C), Nt.BspD6I	Relative cleavage extent, % (30 min, 37°C), ss.BspD6I in complex with Nt.BspD6I
**I**	↓ 5'-CGTGGTCTC**GAGTC**TTCTCAAGGTAC-3' 3'-GCACCAGAG**CTCAG**AAGAGTTCCATG-5' ↑ ↑	74	8 ± 2	6.2 ± 0.7	100	100
**I-A**	↓ 5'-CGTGGTCTC**GAGTC**TT^**X**^CTCAAGGTAC-3' 3'-GCACCAGAG**CTCAG**AA-GAGTTCCATG-5' ↑ ↑	75	12 ± 4	0.5 ± 0.1	81	77
**I-B**	↓ 5'-CGTGGTCTC**GAGTC**TTCT^**X**^CAAGGTAC-3' 3'-GCACCAGAG**CTCAG**AAGA-GTTCCATG-5' ↑ ↑	75	10 ± 3	0.1 ± 0.05	4	11
**I-C**	↓ 5'-CGTGGTCTC**GAGTC**TTCTCA^**X**^AGGTAC-3' 3'-GCACCAGAG**CTCAG**AAGAGT-TCCATG-5' ↑ ↑	75	11 ± 3	3.7 ± 0.2	100	86
**I-D**	↓ 5'-CGTGGTCTC**GAGTC**TT-CTCAAGGTAC-3' 3'-GCACCAGAG**CTCAG**AA_**X**_GAGTTCCATG-5' ↑ ↑	75	14 ± 2	6.5 ± 0.8	100	8
**I-E**	↓ 5'-CGTGGTCTC**GAGTC**TTCTC-AAGGTAC-3' 3'-GCACCAGAG**CTCAG**AAGAG_**X**_TTCCATG-5' ↑ ↑	75	14 ± 3	5.8 ± 0.7	100	11
**I-F**	↓ 5'-CGTGGTCTC**GAGTC**TTCTCA-AGGTAC-3' 3'-GCACCAGAG**CTCAG**AAGAGT_**X**_TCCATG-5' ↑ ↑	75	13 ± 4	0.2 ± 0.05	44	28

The recognition site of R.BspD6I is boldfaced and underlined. ^X^ and _X_ stand for the non-nucleoside insertion in the top and bottom strands, respectively. ↓ and ↑ indicate positions of the hydrolysis of the top and bottom strands, respectively. The relative cleavage extent was calculated as a ratio of the cleavage efficacy of the modified DNA duplex for 30 min at 37°C to the cleavage efficacy of DNA duplex I at the same conditions multiplied by 100. Relative error for cleavage extent did not exceed 15%.

**Table 2:** The characteristics of DNA duplexes used for developing the molecular-decoy approach.

N	DNA duplex	Length, bp	T_m_, ± 1°C
**I-G**	↓ 5'-CGTGGTCTC**GAGTC**TTCT**^Y^**CAAGGTAC-3' 3'-GCACCAGAG**CTCAG**AAGA-GTTCCATG-5'	26	69
**II**	↓ 5'-GATGCTGCCAA**GAGTC**CTCTAGCTTCATAC-3' 3'-CTACGACGGTT**CTCAG**GAGATCGAAGTATG-5'	30	
II*	↓ 5'-GATGCTGCCAA**GAGTC**CTCTAGCTTCATAC-3'-**TAMRA** 3'-CTACGACGGTT**CTCAG**GAGATCGAAGTATG-5'	30	‒
**III**	↓ 5'-GCGTGGTCTC**GAGTC**-TTCTCAAGGTACCTG-3' 3'-CGCACCAG-AG**CTCAG**AAGAGTT-CCATGGAC-5'	30	25
**III-A**	5'-GCGTGGTCTC**GAGTC**-TTCT^X^CAA GGTACCTG-3' 3'-CGCACCAG-AG**CTCAG** AAGA-GTT-CCATGGAC-5'	30	26
**III-B**	5'-GCGTGGTCTC**GAGTC**-TTCT^X^CA^X^AGGTACCTG-3' 3'-CGCACCAG-AG**CTCAG**AAGA-GT-T-CCATGGAC-5'	30	28
**III-C**	5'-GCGTGGTCTC**GAGTC**-TT^X^CT^X^CA^X^AGGTACCTG-3' 3'-CGCACCAG-AG**CTCAG**AA-GA-GT-T-CCATGGAC-5'	30	24
**III-D**	5'-GCGTGGTCTC**GAGTC**-TTCT^Y^CAAGGTACCTG-3' 3'-CGCACCAG-AG**CTCAG**AAGA-GTT-CCATGGAC-5'	30	‒
**IV**	5'-GCGTGGTCTGTGCTA-TTCTCAA GGTACCTG-3' 3'-CGCACCAG-ACACGAT AAGAGTT-CCATGGAC-5'	30	‒

The recognition site of R.BspD6I is boldfaced and underlined.

^X^ ‒ AB insertion

^Y^ ‒ triethylene glycol residue.

↓ indicates the position of Nt.BspD6I hydrolysis. The separated oligonucleotides in the duplexes are marked by a dotted line; the central 14-mer oligonucleotide is double-underlined. Duplex II* has the same sequence as duplex **II** but contains fluorophore TAMRA in the top strand.

### Complex formation by Nt.BspD6I and R.BspD6I with DNA duplexes

Complex formation between a protein and a ^32^P-labeled DNA duplex was carried out at 37°C during 30 min in 10 μl of a buffer consisting of 10 mM Tris-HCl (pH 7.8), 150 mM KCl, 10 mM CaCl_2_, 1 mM DTT, and 0.1 mg/ml BSA; 10 nM DNA duplex and 1–100 nM Nt.BspD6I were used. To form the functional heterodimer of R.BspD6I, a 12-fold excess of the small subunit over Nt.BspD6I was added [51]. Gel electrophoresis was performed in a 7% nondenaturing polyacrylamide gel (PAG) in TBE buffer (89 mM Tris-borate, pH 8.3, 2 mM EDTA) at 15 mA. A Typhoon FLA 9500 was used to obtain autoradiographs of the gels; the autoradiographs were analyzed in the ImageQuant software (GE Healthcare, Great Britain). The yield of a DNA–protein complex (%) was calculated as a ratio of the signal intensities of complexes to the sum of all signals in a lane. The apparent *K*_d_ of the complexes was assumed to be equal to the concentration of Nt.BspD6I at which a half of DNA was associated with the enzyme. The experiments were repeated 3–5 times. The standard error was calculated as SE = s/n^0.5^, where s is a standard deviation, and n is the number of the experiments.

### Hydrolysis of the DNA duplexes by Nt.BspD6I and R.BspD6I

Hydrolysis of DNA duplexes (10 nM) containing 5′-^32^P or 3′-TAMRA (5-carboxytetramethylrhodamine) was carried out at 37°C for 30 min in 10 μl of 10 mM Tris-HCl (pH 7.8) buffer with 150 mM KCl, 10 mM MgCl_2_, 1 mM DTT, 0.1 mg/ml BSA (buffer A) in the presence of 10 nM Nt.BspD6I or a mixture of 10 nM Nt.BspD6I and 120 nM ss.BspD6I. The reaction products were analyzed by gel electrophoresis in a 20% PAG with 7 M urea in TBE buffer at 30 mA followed by visualization using Typhoon FLA 9500. The gel images were analyzed in the ImageQuant program. The extent of DNA cleavage by the enzyme was calculated as a ratio of the signal intensities of a hydrolysis product to the sum of all the signals in a lane. The initial rate of the DNA duplexes’ hydrolysis by Nt.BspD6I was calculated as the tangent of an angle of the initial linear part in the kinetic curve. The experiments were repeated 3–5 times. Standard error was calculated as described above.

### Photo- or thermoregulation of the Nt.BspD6I activity

Nt.BspD6I activity dependence on the UV light irradiation and heating was studied using DNA duplex II* (Table 2); the top strand was 3′-labeled with TAMRA. Hydrolysis was carried out for 5 min at 25, 30, 35, 40, 45, or 50°C in 10 μl of buffer A; 10 nM Nt.BspD6I, 10 nM DNA, and 3 μM inhibitory duplex were used. Mixtures were analyzed as described above for hydrolysis assays. Standard error was less than 12%. To study the influence of UV light on the Nt.BspD6I activity, reaction mixtures without the enzyme were preincubated at a desired temperature under UV light illumination (365 nm) for 10 min. Next, Nt.BspD6I was added, and the reaction was carried out for 5 min during continued exposure to UV light. UV light with a wavelength of 365 nm was generated by the lamp VL-8.MC (Vilber Lourmat, France).

### Hydrolysis of T7 phage DNA by Nt.BspD6I depending on the temperature

T7 phage DNA (5 ng/μl) was hydrolyzed by Nt.BspD6I (12 nM) for 30 min at 20, 25, 30, 35, 40, 45, or 50°C in 10 μl of buffer A in the presence or absence of the competitor DNA duplex (60 μM). After that, 1× SybrGold staining solution (Life Technologies, USA) was added to the reaction mixtures before loading onto a gel, and they were analyzed in a 0.7% agarose gel in the presence of GeneRuler 1 kb DNA Ladder (Thermo Fisher Scientific, USA) as a size marker in TAE buffer (40 mM Tris, 20 mM acetic acid, 1 mM EDTA). The mixtures were analyzed as described above.

## Results and discussion

The aim of this study was to investigate the structural features of R.BspD6I–DNA interactions and to develop DNA-based systems that can temporarily inhibit the catalytic activity of Nt.BspD6I in a stimulus-responsive manner.

### Design of DNA duplexes containing azobenzene residues

**Fig 1:**
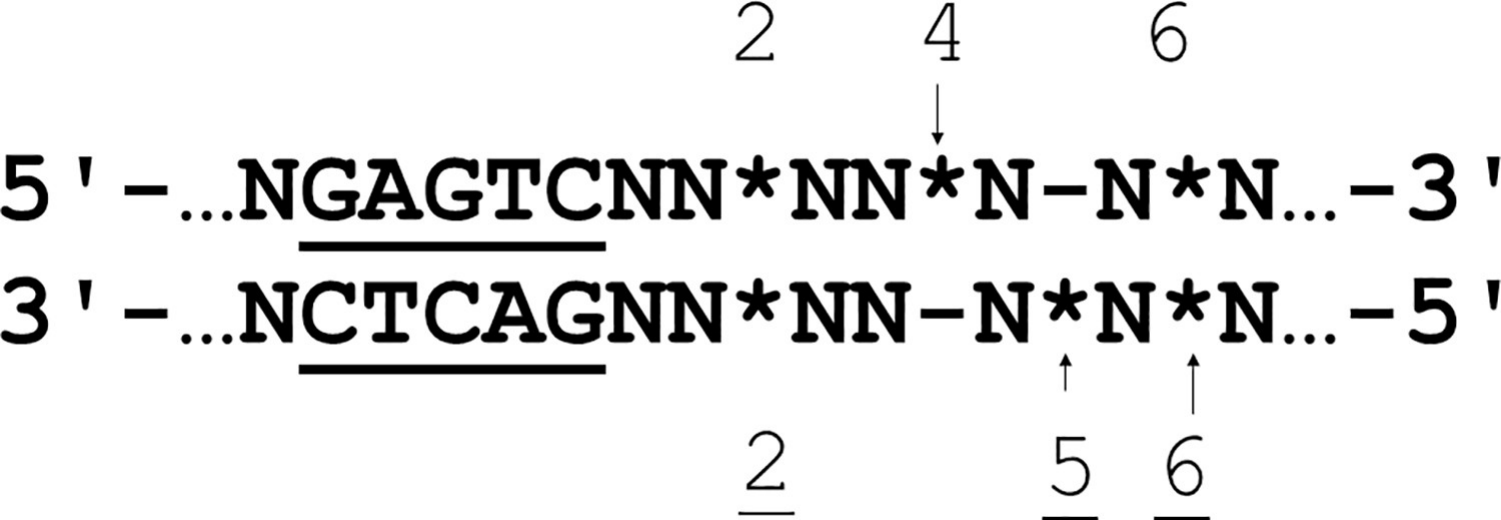
The general scheme of the modification pattern in duplexes I-A to I-F. *Non-nucleoside insertions; arrows indicate the cleavage sites of Nt.BspD6I (top strand) and ss.BspD6I (bottom strand); the longer arrow indicates the major position (6) of hydrolysis in the bottom strand, the shorter one–the minor position of hydrolysis (5). The numbers correspond to the distance in bp between the recognition site and the modification. R.BspD6I recognition site is underlined. Each DNA duplex used in the study contained only a single non-nucleoside insertion.

According to the current structural model of Nt.BspD6I, the N-terminal domain interacts with the binding site in DNA duplexes [50]. Hydrolysis of the top DNA strand occurs in the active center, located in a C-terminal domain of Nt.BspD6I, whereas ss.BspD6I performs hydrolysis of the bottom strand. The modification of the Nt.BspD6I cleavage site should affect the catalytic activity more than the DNA binding. We constructed a number of modified duplexes based on 26-bp R.BspD6I substrate **I** (duplexes **I-A, I-B**, **I-C**, **I-D**, **I-E**, and **I-F** in Table 1). Non-nucleoside residues were inserted as an additional moiety either directly into the sites of hydrolysis of Nt.BspD6I and ss.BspD6I or nearby to study interactions of the subunits with DNA (Fig 1). Besides, we supposed to study the evaluation of the Nt.BspD6I activity in the presence of the DNA duplexes with multiple azobenzene insertions exposed to light. It should be noted that at least two natural nucleotides should separate two AB-containing residues in the design of a DNA sequence for such approach [48] (Table 2, duplexes **III-A**—**III-C**). First of all, it was necessary to investigate the influence of individual modifications on the process of hydrolysis. That is why we introduced a non-nucleoside AB-insertion (S1 Fig) at the position of DNA hydrolysis by Nt.BspD6I (Fig 1, position 4) and between nucleotides located upstream and downstream of the cleavage site (positions 2 and 6). In the bottom strand, the non-nucleoside insertion was located either in one of the cleavage sites of ss.BspD6I (6 –major cleavage site position, or 5 –minor cleavage site position [53]) or two nucleotides downstream of the recognition site (position 2; Fig 1).

### Interactions of DNA duplexes containing azobenzene residues with Nt.BspD6I

First, we demonstrated that AB insertions had no influence on the thermal stability of DNA duplexes **I** and **I-A** to **I-F** by UV-melting (Table 1). Then, we evaluated the binding of Nt.BspD6I to DNA duplexes **I** and **I-A** to **I-F** under visible light (AB in the *trans*-configuration). AB insertions slightly decreased the binding ability of Nt.BspD6I toward the modified DNA duplexes (Table 1). The obtained data confirmed that modifications did not have significant influence on the duplex structure. However, the position of the non-nucleoside insertion slightly affected apparent *K*_d_ of the protein–DNA complex.

**Fig 2:**
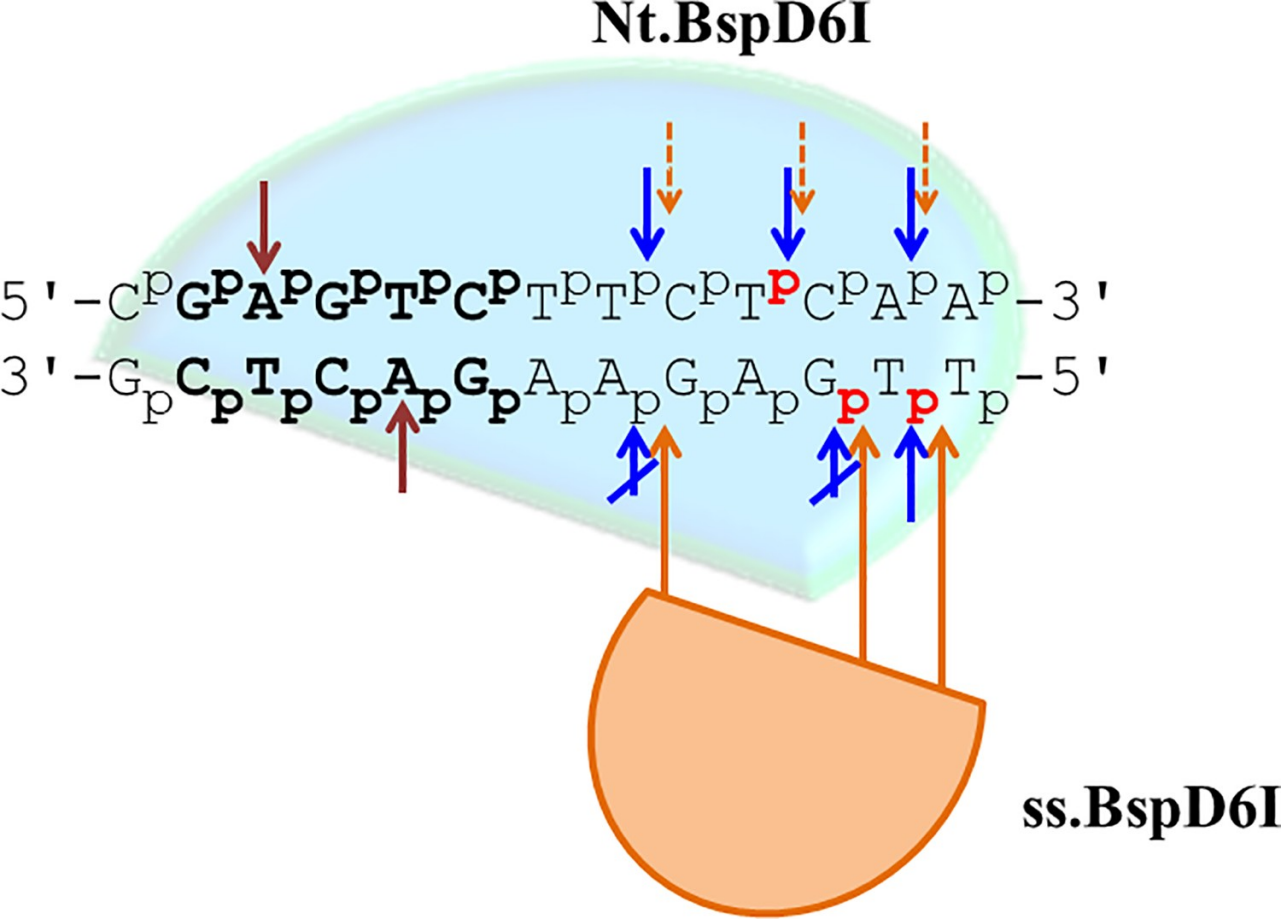
Positions of modifications in R.BspD6I substrate and their influence on the DNA cleavage by Nt.BspD6I and ss.BspD6I. R.BspD6I recognition site is shown in bold. The internucleotide phosphates with phosphodiester bonds undergoing enzyme cleavage are shown in red. Dark red arrows indicate the positions where the presence of N6-methyl-2′-deoxyadenosine in the recognition site of substrate analog blocks the Nt.BspD6I and ss.BspD6I action [51]. The positions where introduction of the non-nucleotide AB-insertion significantly decreases DNA cleavage by Nt.BspD6I are indicated by blue arrows, where influence on initial rate of hydrolysis is minimal–by the crossed blue arrows. Orange short dashed lines show the positions in the top DNA strand where the observed decrease of the ss.BspD6I catalytic activity was insignificant or could be linked to the lowered Nt.BspD6I activity. Modifications with AB-insertions in the bottom DNA strand (positions of modification are indicated by orange long arrows) significantly inhibit the DNA cleavage by ss.BspD6I. The AB-insertions 5 and 6 bp downstream the recognition sequence are localized directly in the ss.BspD6I cleavage sites.

Next, hydrolysis of ^32^P-labeled modified DNA duplexes **I-A** to **I-F** by Nt.BspD6I was studied as described earlier [51]. Nonetheless, location of the modification had an influence on the initial cleavage rate (Table 1). The initial cleavage rates (*v*_0_) of duplexes **I-D** and **I-E** are similar to *v*_0_ of non-modified duplex **I**. Therefore, the AB-insertion two nucleotides downstream from the recognition site (duplex **I-D**) in the bottom strand and in one of the ss.BspD6I hydrolysis sites (duplex **I-E**) are not essential for Nt.BspD6I interaction with the DNA. DNA duplex **I-C** contains an AB insertion in the top strand (six nucleotides downstream of the Nt.BspD6I hydrolysis site). The *v*_0_ of this duplex was lower as compared to unmodified duplex **I** (Table 1). Thus, despite the distal location of this modification, the enzyme was able to interact with this region (Fig 2). The *v*_0_ of DNA duplex **I-A** (containing an AB-insertion in the top strand located between the recognition site and the cleavage site of Nt.BspD6I) was dramatically decreased (approximately 12-fold) in comparison with *v*_0_ of the unmodified DNA duplex **I** (Table 1).Obviously, unmodified sugar-phosphate backbone structure of this position (Fig 1) is necessary for effective Nt.BspD6I functioning. Insertion of the modification into the Nt.BspD6I cleavage site (DNA duplex **I-B**) prevented the hydrolysis by Nt.BspD6I. Thus, DNA duplex **I-B** was chosen as a candidate for the Nt.BspD6I inhibitor because Nt.BspD6I bound to it effectively and barely hydrolyzed it. Duplex **I-F** containing AB-insertion in the main cleavage site of ss.BspD6I (Fig 1, position 6) had a low *v*_0_ and extent of DNA hydrolysis in this case did not exceed 50% (Table 1). We propose that Nt.BspD6I interacts with the sugar-phosphate backbone of the bottom strand (6 nucleotides downstream from the recognition site) to coordinate ss.BspD6I on the DNA. This event is then able to stimulate DNA hydrolysis by ss.BspD6I of the bottom strand (see below).

### Interactions of the DNA duplexes containing azobenzene moieties with the small subunit of restriction endonuclease BspD6I

**Fig 3:**
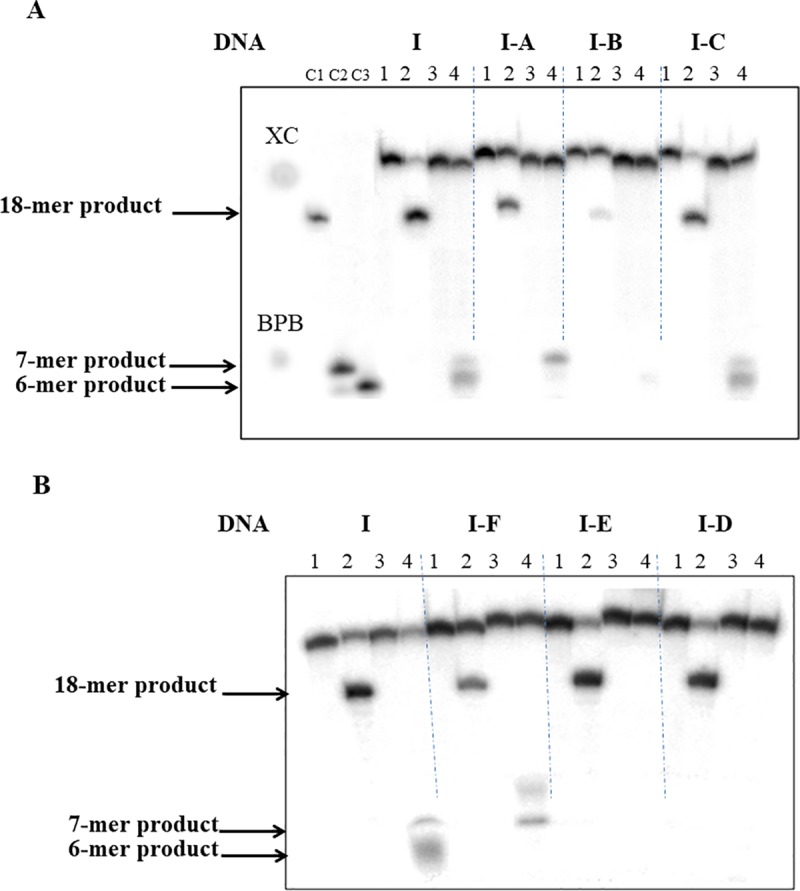
**Analysis of the cleavage products of DNA duplexes (10 nM) I-A to I-C (A) and I-D to I-F (B) compared to the hydrolysis of control DNA duplex I by Nt.BspD6I or ss.BspD6I in the presence of Nt.BspD6I in 20% PAG with 7 M urea**. Lanes 1: initial DNA (^32^P-labeled top strand), lanes 2: hydrolysis of the DNA duplex (^32^P-labeled top strand) by Nt.BspD6I (10 nM), lanes 3: initial DNA (^32^P-labeled bottom strand), lanes 4: the hydrolysis of the DNA duplex (^32^P-labeled bottom strand) by ss.BspD6I (120 nM) in the presence of Nt.BspD6I (10 nM). XC: xylene cyanol, BPB: bromophenol blue. C1, C2, and C3: control samples: ^32^P-labeled oligonucleotides with the lengths of 18, 7, and 6 nucleotides, respectively.

Next, we studied hydrolysis of DNA duplexes containing AB **(I-A to I-F**) by ss.BspD6I in the presence of Nt.BspD6I. The R.BspD6I heterodimer was formed by mixing Nt.BspD6I and ss.BspD6I at the ratio of 1:12 (we had demonstrated previously that the enzyme has a maximal activity under these conditions [51]). We compared the influence of the AB-insertion location in the DNA duplex on Nt.BspD6I activity and on ss.BspD6I functioning in the presence of Nt.BspD6I (Table 1). Introduction of the non-nucleoside AB-insertion at position 2 of the top strand (DNA duplex **I-A**) and at position 6 opposite the ss.BspD6I cleavage site (DNA duplex **I-C**) almost had no influence on DNA cleavage by Nt.BspD6I and led to a decrease in the hydrolysis efficiency of ss.BspD6I by 15–20% (Fig 3A). The non-hydrolyzable analog of the Nt.BspD6I substrate, DNA duplex **I-B** (AB-insertion in the cleavage site of Nt.BspD6I), was not hydrolyzed by ss.BspD6I either (Fig 2). For DNA duplexes **I, I-B** and **I-C** we observed the prevalence of a 6-mer product of the hydrolysis by ss.BspD6I (in the presence of Nt.BspD6I). DNA duplex **I-A** (AB-insertion located at position 2) was an exception because the main product of its hydrolysis was a 7-mer oligonucleotide: the ratio of the 7-mer to 6-mer product was 4:1.

AB-insertions in the bottom strand close to the recognition site at position 2 (DNA duplex **I-D**) or at position 5 (minor ssBspD6I cleavage site, DNA duplex **I-E**) allowed Nt.BspD6I to hydrolyze the corresponding duplexes as efficiently as unmodified substrate **I.** In contrast, modification of these positions inhibited significantly the catalytic activity of ss.BspD6I. DNA duplex **I-F** can be considered as a “bad” substrate for Nt.BspD6I; this result therefore should directly affect the ss.BspD6I activity. Indeed, modification of the main ss.BspD6I cleavage site (position 6, DNA duplex **I-F**) led to a significant decrease in the hydrolytic efficiency of the bottom DNA strand (Fig 3B). In the case of DNA duplex **I-F**, mobility of the bottom strand cleavage products (5′-GTACCT**x**T-3′ and 5′-GTACCT**x**-3′, where **x** is an AB-insertion) in the gel was slower because they contained the AB modification.

According to the data in Table 1, the catalytic activity of Nt.BspD6I is necessary for the ss.BspD6I functioning. Nonetheless, modification of the bottom strand at position 2 (DNA duplex **I-D**) or 5 (DNA duplex **I-E**) inhibited the ss.BspD6I activity, whereas Nt.BspD6I hydrolyzed these DNA duplexes effectively. Thus, it was shown for the first time that for effective ss.BspD6I functioning not only its cleavage sites in the bottom strand should be intact but also the region close to the recognition site (position 2). We propose that in addition to the “correct” Nt.BspD6I conformation in complex with DNA, ss.BspD6I should interact with sugar-phosphate backbone nearby the recognition site to form an active Nt.BspD6I-ss.BspD6I-DNA complex. This hypothesis is in agreement with the results obtained in the analysis of Nt.BspD6I interaction with methylated DNA duplexes [51] and properties of the Nt.BspD6I variants [52].

### The development and optimization of modified DNA duplexes for regulation of the Nt.BspD6I activity

**Fig 4:**
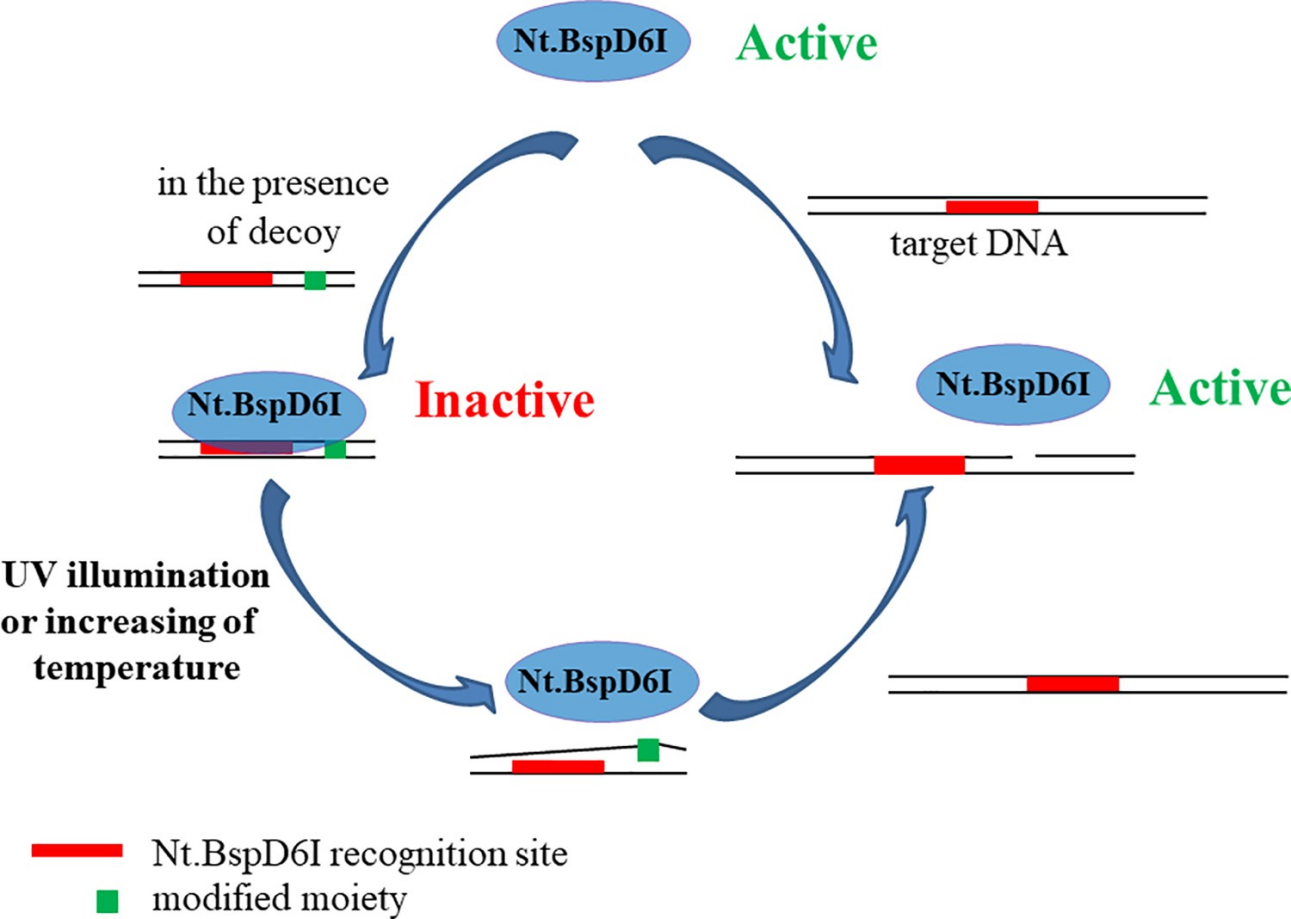
The “molecular decoy” approach developed in this study. Based on the results obtained, it is proposed to use short non-hydrolyzable DNA duplexes containing modification in the position of the Nt.BspD6I hydrolysis as reversible decoys. In the beginning, the excess of the DNA decoy blocks the DNA binding and catalytic center of the enzyme and after UV illumination or heating DNA duplexes should dissociate. This will cause dissociation of DNA-protein complexes and the restoration of the enzymatic activity towards target (e.g. genomic) DNA.

In this study, we found a number of positions in the DNA substrate crucial for interactions with R.BspD6I components. Accordingly, we proceeded to develop a general approach to improving applicability of NEases in assays and genome editing by reversible inhibition. The idea was to use nonhydrolyzable DNA duplexes containing AB insertions as reversible decoys for Nt.BspD6I (Fig 4). Preformed inactive decoy–enzyme complexes should preclude nonspecific hydrolysis, while UV illumination or heating will cause dissociation of complexes thus restoring the NEase activity. Previously, we have demonstrated that Nt.BspD6I can bind with short unmodified DNA duplexes (13 and 15 bp) with the recognition site of Nt.BspD6I and only 4 bp downstream the recognition site, i.e. they did not contain a phosphodiester bond that was cleaved by the enzyme [56]. Nevertheless, a huge excess of such duplexes was needed to inhibit the catalytic activity of Nt.BspD6I owing to poor affinity. Here we performed thorough optimization of the duplex structure to improve the inhibition efficacy. First, we proposed that the oligonucleotide decoy should contain both a binding site and a site of hydrolysis because of the two-domain structure of Nt.BspD6I. Second, thermal stability of the inhibitory duplex should be thoroughly tuned to ensure the initial robust DNA binding to Nt.BspD6I and fast DNA duplex dissociation after heating and/or UV light irradiation that causes effective enzyme activation due to disruption of the DNA–protein complex.

We started with 26-bp duplex **I-B** (Table 1) bearing one AB-insertion at the Nt.BspD6I cleavage site as a decoy and 30-bp duplex **II** as a model target (Table 2). In the presence of 100-fold molar excess of duplex **I-B**, hydrolysis of the substrate at 37°C was inhibited by 90% (S2 Fig), but UV irradiation had only a minimal effect (data not shown) owing to high duplex stability and insufficient duplex destabilization by changes in a single AB residue (Table 1). Consequently, we dissected the top strand of duplex-decoy into three oligonucleotides, and the bottom strand into two oligonucleotides (duplex **III**, Table 2). This duplex with gaps in each strand manifested noncooperative melting at low temperature, in the range from 15 to 40°C (S3 Fig).

The main component of duplex **III** is the 14-mer oligonucleotide in the top strand with a single or multiple AB-insertions (duplexes **III-A**, **III-B**, and **III-C**). The AB-insertion was incorporated into the Nt.BspD6I cleavage site of the 14-mer oligonucleotide (duplex **III-A**), and this insertion prevented hydrolysis of the duplex (S4 Fig). To enhance the influence of the AB photo isomerization on the stability of the gapped duplex, additional AB-insertions were introduced into the 14-mer oligonucleotide at position 6 (duplex **III-B**) or positions 2 and 6 (duplex **III-C**) relative to the recognition site (Fig 1). Duplexes **III**, **III-A**, **III-B**, and **III-C** dissociated in the same temperature range: 15–40°C (S3 Fig).

**Fig 5:**
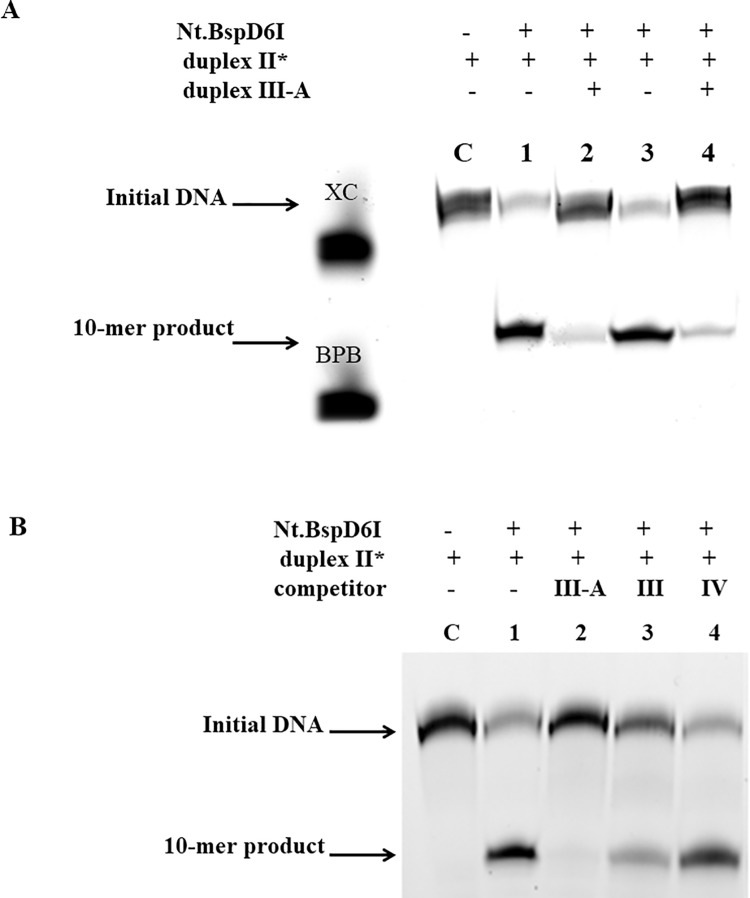
Analysis of cleavage of 30-bp substrate II* by Nt.BspD6I in the presence of the DNA competitors (20% PAG with 7 M urea). The reactions were incubated for 30 min at 25°C. **A**. Analysis of the Nt.BspD6I (10 nM) activity in the presence of duplex **III-A** at its different concentrations. Lane C: initial duplex II* (10 nM). Lanes 1, 3: hydrolysis of DNA duplex II* by Nt.BspD6I. Lanes 2, 4: hydrolysis of DNA duplex II* by Nt.BspD6I in the presence of a 200-fold or 300-fold excess of DNA duplex **III-A**, respectively. **B**. Analysis of hydrolysis of DNA duplex II* (10 nM) by Nt.BspD6I (10 nM) in the presence of the 300-fold excess of duplex **III, III-A**, or **IV**. Lane C: initial DNA fragment (10 nM), lane 1: hydrolysis of DNA duplex II* by Nt.BspD6I.

First, we checked the hydrolysis of duplexes **III** and **III-A** by Nt.BspD6I using a ^32^Р-labeled internal 14-mer oligonucleotide (S4 Fig). Duplex **III-A** was stable in the presence of Nt.BspD6I; therefore, we used it as a temporary decoy for the enzyme. Then, we studied hydrolysis of duplex II* by Nt.BspD6I in the presence of various excesses of duplex **III-A** over the substrate (Fig 5). Only a 300-fold excess of duplex **III-A** could inhibit the Nt.BspD6I activity by 90% at 25°C (Fig 5A). Probably the reason is low stability of duplex **III-A** (melting temperature around 25°C). To confirm specificity of the Nt.BspD6I inhibition by duplex **III-A** at 25°C, we demonstrated that duplex **IV** without an Nt.BspD6I recognition site could not inhibit the hydrolysis of the target DNA (Fig 5B). The excess of native duplex **III** with the Nt.BspD6I recognition site also suppressed hydrolysis of substrate II* by Nt.BspD6I, but less effectively than duplex **III-A** did owing to its own hydrolysis. Thus, duplex **III-A** was shown to be the most effective inhibitor of the Nt.BspD6I activity.

### Evaluation of the Nt.BspD6I activity in the presence of the azobenzene-containing DNA duplexes exposed to light

We studied kinetics of the substrate II* hydrolysis by Nt.BspD6I in the presence of a 300-fold excess of a DNA duplex **III-A**, **III-B**, or **III-C** under UV light illumination at 365 nm. In control experiments, UV light was shown to have no effect on the Nt.BspD6I activity (S5 Fig). No significant influence of UV light on the initial rates of substrate’s hydrolysis was observed at 25°C (S6 Fig). Heating of the reaction mixture led to the dissociation of duplex **III-A**; accordingly, UV-driven regulation was not applicable to this duplex (S7A Fig). Nonetheless, we observed effects opposite to the proposed one. Duplexes **III-B** and **III-C** inhibited Nt.BspD6I more effectively under UV light irradiation than in darkness, and the strongest effect was observed for DNA duplex **III-C** with three AB-insertions. *Cis*-configuration of the azobenzene in duplexes **III-B** and **III-C** may promote DNA bending and thus stimulates Nt.BspD6I binding [51]. A maximal difference in the hydrolysis efficiency was observed at 40°C (S7B Fig).

On the other hand, we observed distinct temperature-dependent regulation of the Nt.BspD6I activity. The 300-fold excess of duplex **III-A** over the substrate almost completely blocked Nt.BspD6I activity in the temperature range of 25–30°C, whereas at 45°C, activity of Nt.BspD6I was restored due to the dissociation of a modified DNA duplex. Therefore, we found the option to use DNA duplexes to switch the Nt.BspD6I activity on or off by temperature variation. Previously, we estimated the possibility of thermoregulation of the REase’s activity by means of DNA fragments using conjugates of REase SsoII with oligodeoxyribonucleotides [57].

### The DNA duplex with a triethylene glycol residue for the temperature-dependent regulation of the Nt.BspD6I activity

**Fig 6:**
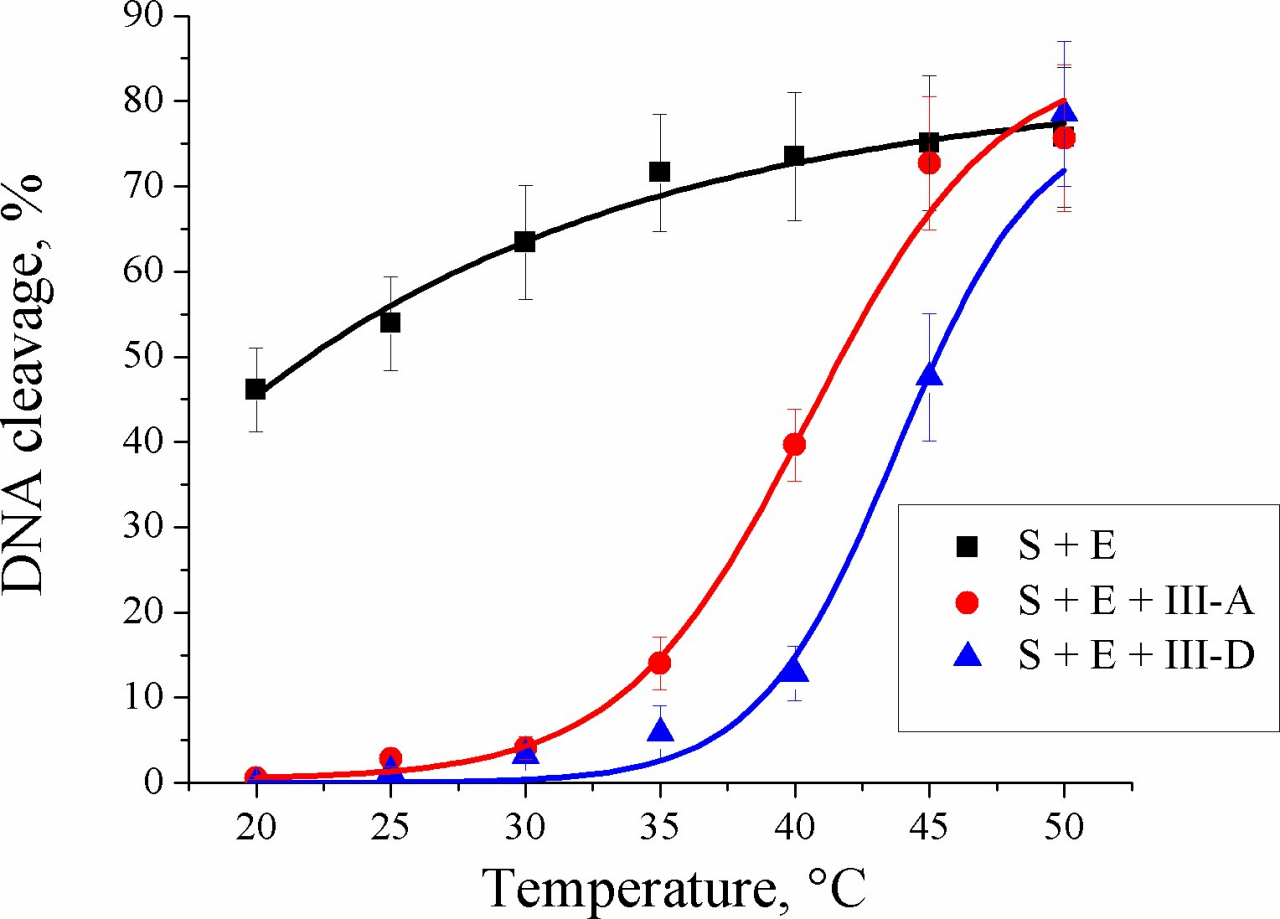
Temperature dependence of the hydrolysis efficacy of target DNA II* (S, 10 nM) by Nt.BspD6I (E, 10 nM) in the absence (black curve) and in the presence of the 300-fold excess of DNA duplex III-A (red curve) or III-D (blue curve). The reactions were allowed to proceed for 5 min.

Temperature-dependent inhibition of Nt.BspD6I by modified duplexes with azobenzene prompted us to use a more suitable triethylene glycol residue in the hydrolysis site of Nt.BspD6I. It has been shown previously that introduction of oligoethylene glycol linkers results in an independent thermodynamic behavior of the connected parts in oligonucleotides [58]; this principle has been used to develop telomerase inhibitors [59] and molecular beacons [60]. Triethylene glycol-modified duplex **I-G** has lower melting temperature in comparison with unmodified duplex **I** or the azobenzene-containing duplex **I-B** (Tables 1 and 2) and was poorly hydrolyzed by Nt.BspD6I (less than 20% in 30 min). Thus, we synthesized duplex **III-D** and compared its inhibitory activity toward Nt.BspD6I with that of duplex **III-A** in the temperature range 25–50°C (Fig 6). At 25–30°C, duplexes **III-A** and **III-D** effectively inhibited DNA hydrolysis by Nt.BspD6I. By contrast, at 40–45°C, DNA duplex **III-D** inhibited the Nt.BspD6I activity more effectively than did DNA duplex **III-A**; this phenomenon could be a result of DNA bending [61], which can promote Nt.BspD6I binding [51].

**Fig 7:**
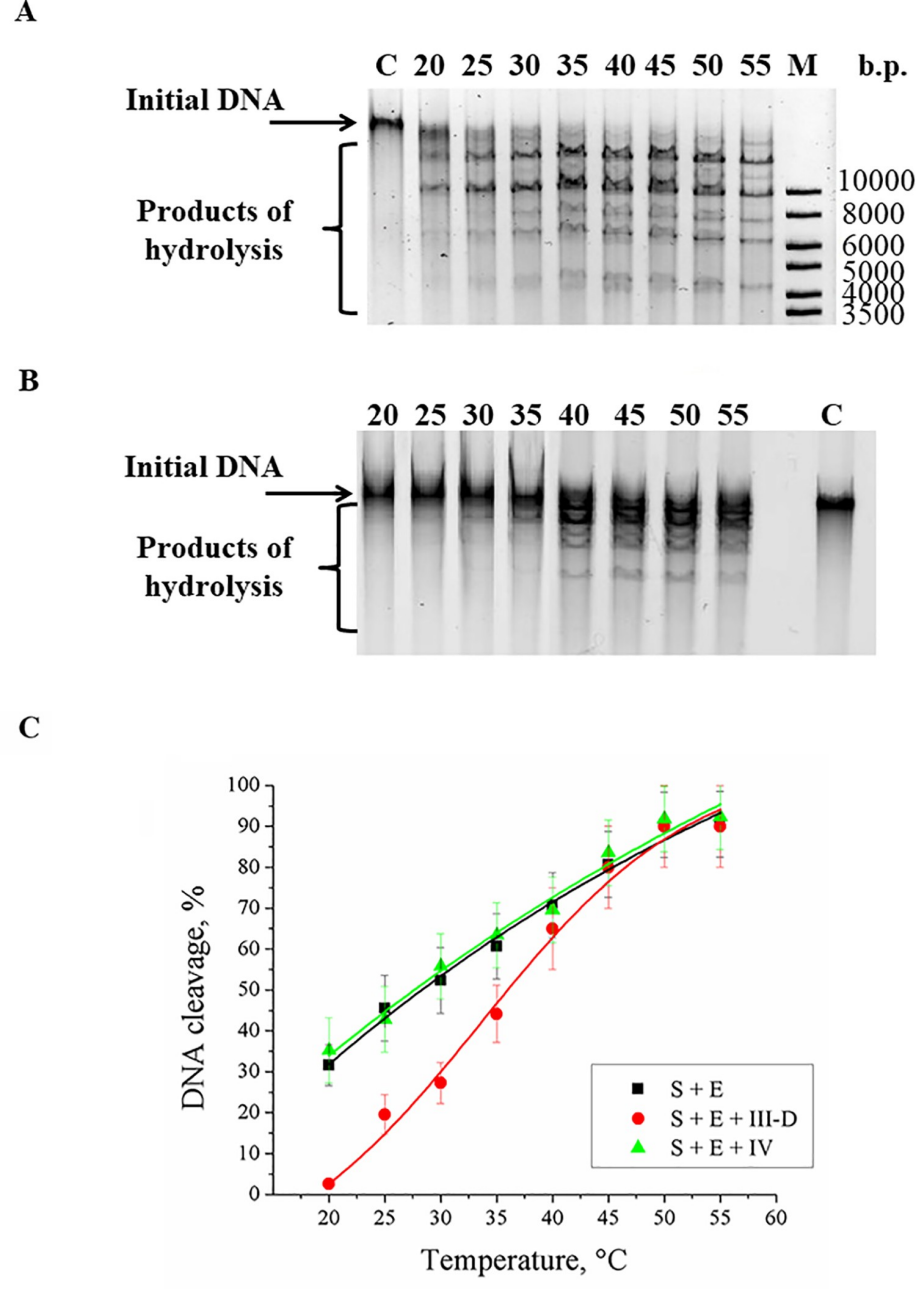
Temperature dependence of the hydrolysis efficacy of T7 phage DNA (S) by Nt.BspD6I (E) in the absence or presence of inhibitor III-D or nonspecific duplex IV. A and B: analysis in a 0.7% agarose gel of T7 phage DNA cleavage in the absence or presence of inhibitor **III-D**, respectively. The temperatures at which the reaction was carried out are shown above the lanes. DNA was detected by means of fluorescent signals from SYBR Gold. Lane C: the initial DNA, M: DNA ladder. C: a graph of temperature dependence of T7 phage DNA cleavage by Nt.BspD6I in the absence of a competitor (black curve) and in the presence of DNA duplex **III-D** (red curve) or **IV** (green curve).

NEases can cut DNA only when two oppositely directed recognition sites are located close to each other in individual DNA strands. T7 phage DNA contains 115 recognition sites for Nt.BspD6I, but only four of them (5′-GAGTC-3′/3′-CTCAG-5′) are located close to each other [62]. After such promising results with model oligonucleotides, we analyzed hydrolysis of long T7 phage DNA (40 kbp) by Nt.BspD6I in the presence or absence of duplex **III-D** (Fig 7A and 7B). We increased concentration of duplex **III-D** to 60 μM in contrast to the previous experiments because it is known that affinity of restriction endonucleases for DNA is higher for longer substrates [63]. Addition of duplex **III-D** into the reaction mixture effectively blocked the Nt.BspD6I activity at 20–25°C (Fig 7B), whereas nonspecific duplex **IV** hardly affected the Nt.BspD6I activity (Fig 7C). At the same time, the Nt.BspD6I activity was completely restored at 45°C even in the presence of a significant excess of DNA duplex **III-D**. We want to emphasize that the switching temperature of the inhibitory DNA duplex can be easily tuned by variation of the oligonucleotide length; consequently, our system can be adapted to various applications.

## Conclusions

A thorough study of R.BspD6I-DNA substrate interactions using modified oligonucleotides with azobenzene residue was conducted. For the first time, it was demonstrated that for effective ss.BspD6I functioning not only the cleavage sites in the bottom strand should not contain modification but also the region close to the recognition site. Nt.BspD6I was found to interact with the sugar-phosphate backbone of the bottom strand. We propose that Nt.BspD6I coordinates ss.BspD6I on the DNA, and this event drives the hydrolysis of the bottom strand. These results support the hypothesis that a “correct” conformation of Nt.BspD6I–DNA complex and formation of additional ss.BspD6I-DNA contacts are needed for ss.BspD6I functioning.

Results of Nt.BspD6I interaction with DNA duplexes containing AB-insertions allowed to design a series of modified DNA duplexes containing recognition and hydrolysis sites of Nt.BspD6I. Among them, duplex **III-D** with intrastrand triethylene glycol linkage at the site of Nt.BspD6I hydrolysis showed the best inhibition at 20–25°C with an effective release of Nt.BspD6I after heating to 45°C. Thus, proof-of-concept experiments were conducted and the “reversible molecular decoy” approach to temperature-dependent regulation of NEase activity by modified oligonucleotides was validated. Hopefully, this approach will facilitate the development of novel bioanalytical assays and DNA amplification with strand displacement based on NEases and will increase applicability of fused NEs to genome editing. Off-target effects in CRISPR-Cas9 editing result from low sensitivity to non-complementary bases in gRNA genome DNA duplexes [64–66]. Recently thermostable Cas9 proteins were described [67,68] and applied for gene editing at increased temperatures. In these conditions the off-target problem decreases due to higher mismatch sensitivity. It is possible to construct fusion thermostable Cas9 proteins with NEs to improve genome editing.

## Supporting information

S1 Fig The non-nucleoside D-threoninol azobenzene moiety in a DNA strand. B_1_ and B_2_: heterocyclic bases.
(TIF)

S2 Fig Analysis of 30-bp substrate II cleavage by Nt.BspD6I in the presence of DNA duplex I-B. An autoradiograph of 20% PAG containing 7 M urea. The reaction was allowed to proceed for 30 min at 37°C. Lane C corresponds to the initial DNA (^32^P-labeled top strand of DNA duplex **II**); other lanes correspond to hydrolysis of substrate **II** (10 nM) by Nt.BspD6I (10 nM) in the presence of the DNA duplex **I-B** (the concentrations varied from 0 to 1 mM).
(TIF)

S3 Fig Differential melting curves of DNA duplexes III, III-A, III-B, and III-C: dependence of first-order derivatives of the solutions’ optical density on the temperature. Concentrations of the DNA duplexes were 0.4–0.5 μM. The azobenzene moiety was in the *trans*-configuration.
(TIF)

S4 Fig Analysis of the cleavage of DNA duplexes III and III-A and 14-bp duplex V: 5′-TCGAGTCTTCTCAA-3′/3′-AGCTCAGAAGAGTT-5′, in the presence of Nt.BspD6I. An autoradiograph of 20% PAG containing 7 M urea. The reactions were carried out at 25°C for 3 h. Lanes 1, 3 and 5 are initial DNA duplexes **III**, **III-A** and **V**, respectively (10 nM duplex, ^32^P-labeled 14-mer oligonucleotide); lanes 2, 4, 6: the hydrolysis of DNA duplexes **III**, **III-A** and **V** by Nt.BspD6I (10 nM), respectively. XC: xylene cyanol, BPB: bromophenol blue.
(TIF)

S5 Fig Time dependence of the hydrolysis efficacy of target DNA II* (S, 10 nM) by Nt.BspD6I (E, 10 nM) upon exposure to UV light or without the exposure. The experiments were carried out three times. The average values of the cleavage extent are plotted; error did not exceed 12% of the presented value.
(TIF)

S6 Fig Time dependence of the hydrolysis efficacy of target DNA II* (10 nM) by Nt.BspD6I (10 nM) in the presence of the 300-fold excess of DNA duplex III-A during exposure to UV light (red circles) or without the exposure (black squares). The experiments were conducted at least three times. The average values of the cleavage extent are plotted; error did not exceed 12% of the presented value.
(TIF)

S7 Fig Temperature dependence of the hydrolysis efficacy of target DNA II* (S, 10 nM) by Nt.BspD6I (E, 10 nM) in the presence of the 300-fold excess of different inhibitory duplexes during exposure to UV light or without the exposure. The hydrolysis reactions were allowed to proceed for 5 min. The experiments were carried out at least three times. The average values of the cleavage extent are plotted; error did not exceed 12% of the presented value. **A.** Analysis of the Nt.BspD6I activity in the presence of duplex **III-A**; **B.—**in the presence of duplexes **III-B** and **III-C.**
(TIF)
