# Supplementary figures and images for: A study on endonuclease BspD6I and its stimulus-responsive switching by modified oligonucleotides
Source: PLoS One. 2018 Nov 26;13(11):e0207302. doi: 10.1371/journal.pone.0207302 (PMC6261011; doi:10.1371/journal.pone.0207302)

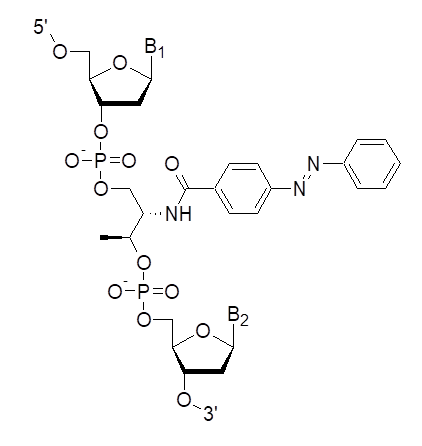

Supplement: S1 Fig — B1 and B2: heterocyclic bases. (TIF) [file pone.0207302.s001.tif]

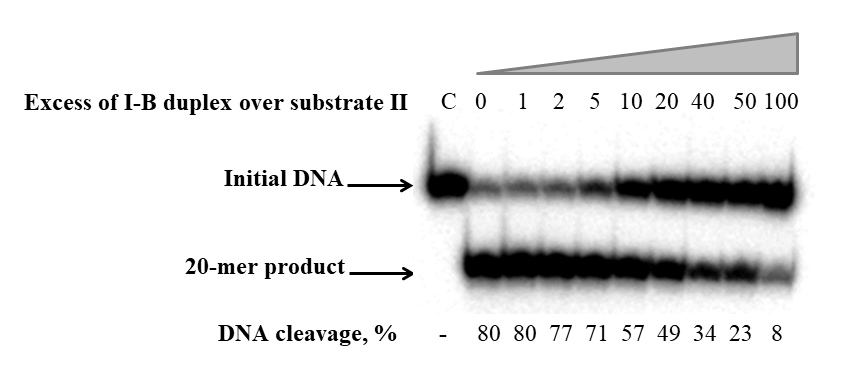

Supplement: S2 Fig — An autoradiograph of 20% PAG containing 7 M urea. The reaction was allowed to proceed for 30 min at 37°C. Lane C corresponds to the initial DNA (32P-labeled top strand of DNA duplex II); other lanes correspond to hydrolysis of substrate II (10 nM) by Nt.BspD6I (10 nM) in the presence of the DNA duplex I-B (the concentrations varied from 0 to 1 mM). (TIF) [file pone.0207302.s002.tif]

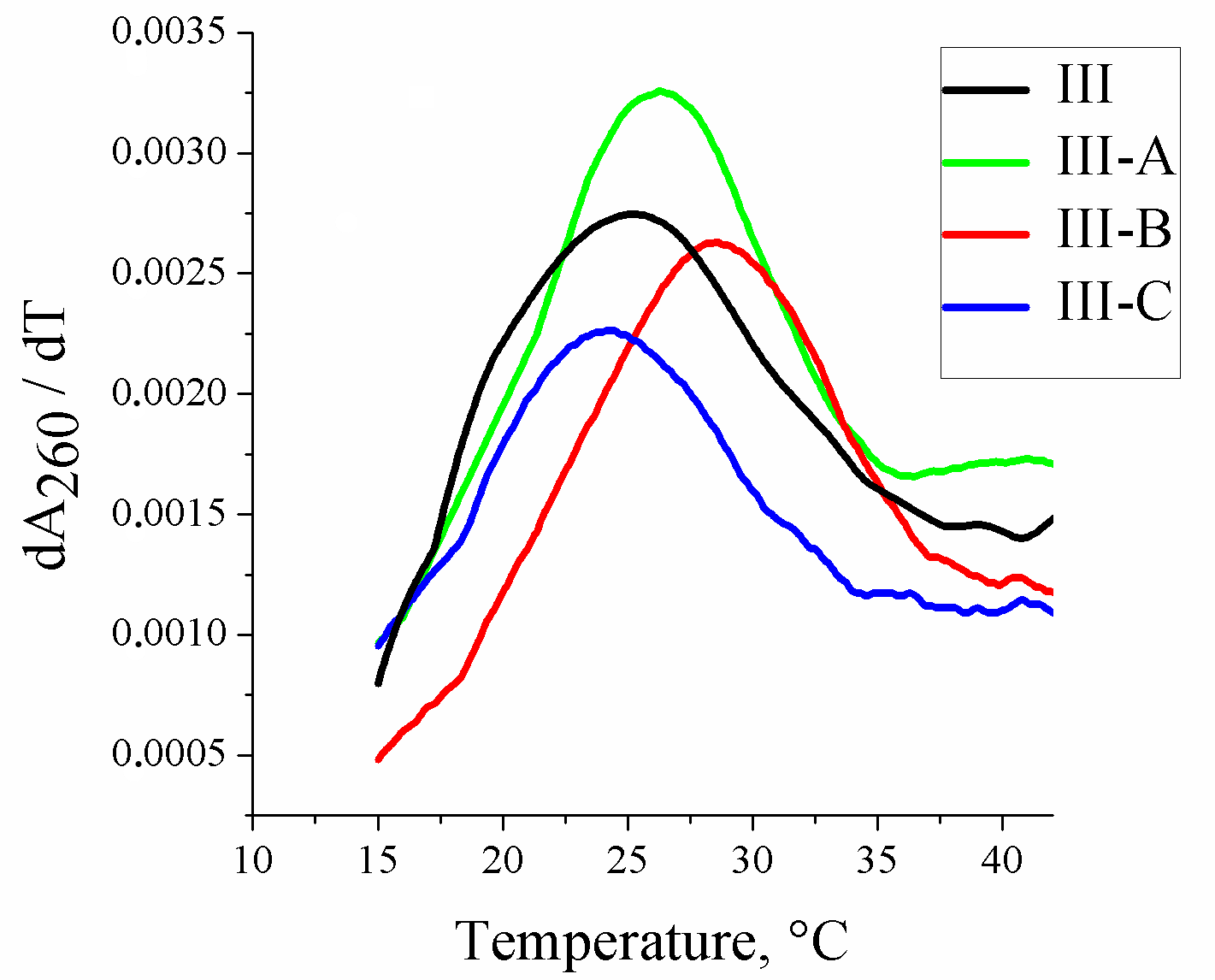

Supplement: S3 Fig — Concentrations of the DNA duplexes were 0.4–0.5 μM. The azobenzene moiety was in the trans-configuration. (TIF) [file pone.0207302.s003.tif]

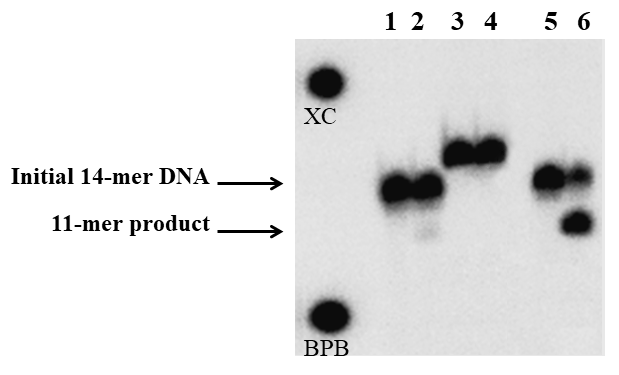

Supplement: S4 Fig — An autoradiograph of 20% PAG containing 7 M urea. The reactions were carried out at 25°C for 3 h. Lanes 1, 3 and 5 are initial DNA duplexes III, III-A and V, respectively (10 nM duplex, 32P-labeled 14-mer oligonucleotide); lanes 2, 4, 6: the hydrolysis of DNA duplexes III, III-A and V by Nt.BspD6I (10 nM), respectively. XC: xylene cyanol, BPB: bromophenol blue. (TIF) [file pone.0207302.s004.tif]

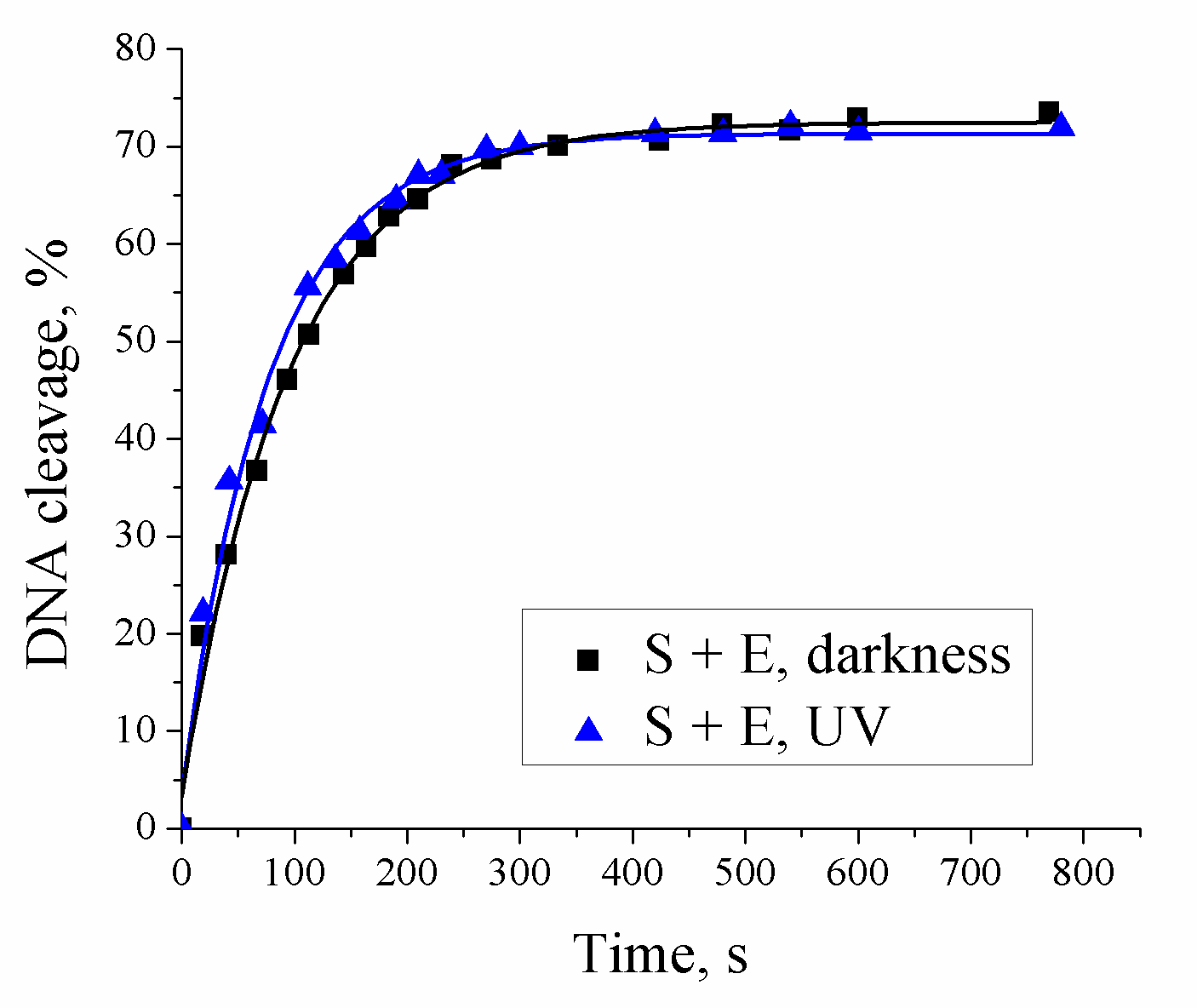

Supplement: S5 Fig — The experiments were carried out three times. The average values of the cleavage extent are plotted; error did not exceed 12% of the presented value. (TIF) [file pone.0207302.s005.tif]

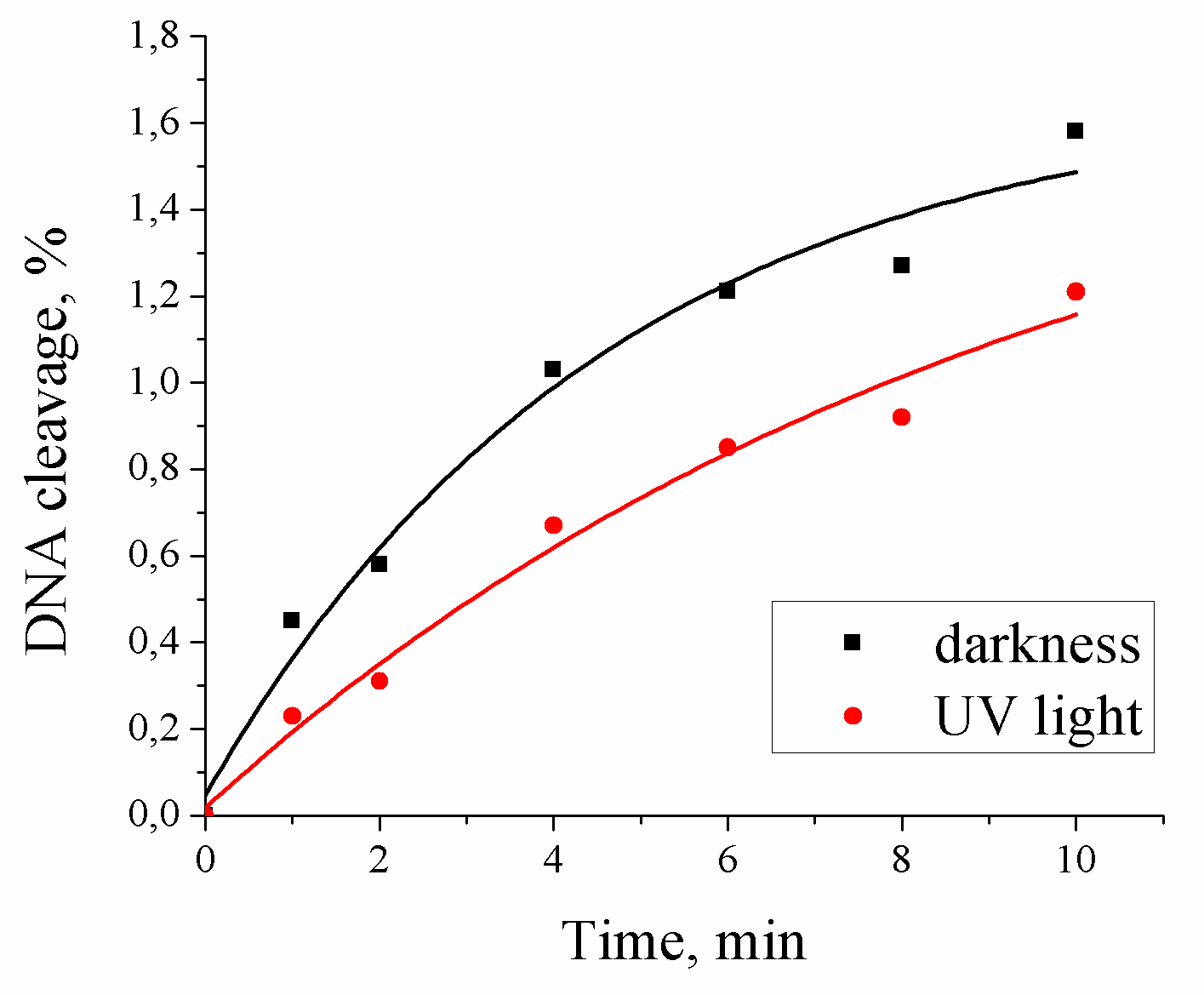

Supplement: S6 Fig — The experiments were conducted at least three times. The average values of the cleavage extent are plotted; error did not exceed 12% of the presented value. (TIF) [file pone.0207302.s006.tif]

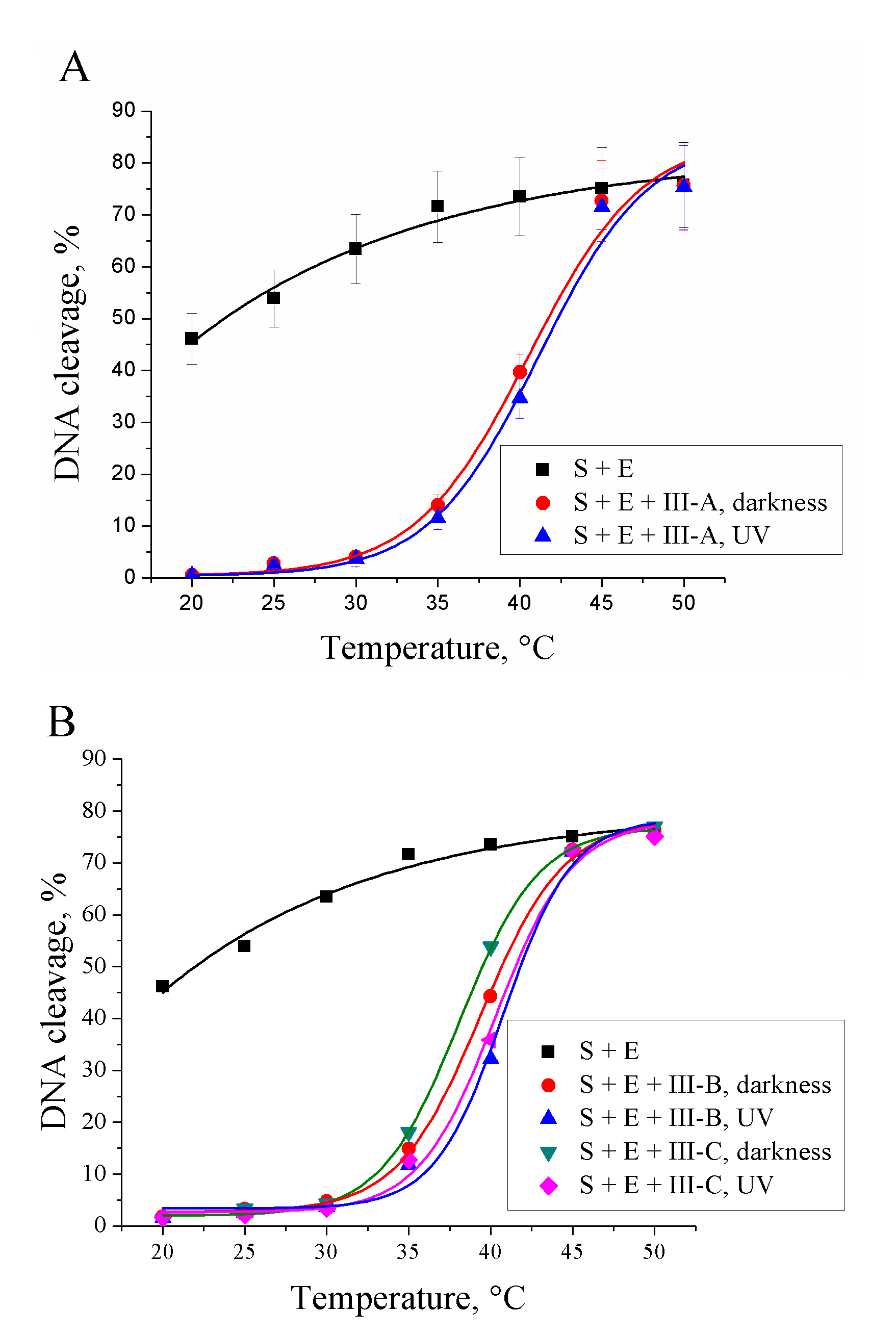

Supplement: S7 Fig — The hydrolysis reactions were allowed to proceed for 5 min. The experiments were carried out at least three times. The average values of the cleavage extent are plotted; error did not exceed 12% of the presented value. A. Analysis of the Nt.BspD6I activity in the presence of duplex III-A; B.—in the presence of duplexes III-B and III-C. (TIF) [file pone.0207302.s007.tif]
